# *Aldo-ketoreductase 1* (*AKR1*) improves seed longevity in tobacco and rice by detoxifying reactive cytotoxic compounds generated during ageing

**DOI:** 10.1186/s12284-017-0148-3

**Published:** 2017-04-13

**Authors:** Kodadinne Narayana Nisarga, Ramu S Vemanna, Babitha Kodekallu Chandrashekar, Hanumantha Rao, Amaranatha Reddy Vennapusa, Ashwini Narasimaha, Udayakumar Makarla, Mohan Raju Basavaiah

**Affiliations:** Department of Crop Physiology, University of Agriculture Sciences, GKVK, Bengaluru, 560065 India

**Keywords:** Oxidative stress, Seed viability, Protein glycation, Ageing, Carbonyl stress, Transgenics

## Abstract

**Background:**

Maintenance of seed viability is an important factor for seedling vigour and plant establishment. Lipid peroxidation mediated reactive carbonyl compounds (RCC’s) and non-enzymatic modifications of proteins through Maillard and Amadori products reduce seed viability and seedling vigour.

**Results:**

In this study, the relevance of RCCs on genotypic variation in rice seed viability and overexpression of an aldo-ketoreductase (AKR1) enzyme that detoxify cytotoxic compounds to improve seed viability and vigour was studied. Physiological and biochemical approaches were integrated to quantify the variation in seed viability and seedling vigour in rice genotypes after exposing to ageing treatment. AKR1 was overexpressed in a susceptible rice genotype and tobacco to study the relevance of reduced RCC’s on seed viability and seedling vigour. The glycation and lipid peroxidation compounds accumulated after accelerated ageing treatments in rice genotypes. The accumulation of malondialdehyde, methyl glyoxal, Maillard and Amadori products affected the seed viability and germination as they showed a significant negative relationship. The transgenic rice and tobacco seeds expressing *AKR1* showed lower levels of cytotoxic compounds and glycation products that resulted in improved seed viability and seedling vigour in rice and tobacco.

**Conclusions:**

The study demonstrates that, reactive cytotoxic compounds affect the seed viability during storage. Detoxification of reactive cytotoxic compounds by Aldo-keto reductases is one of the mechanisms to improve the seed longevity during storage.

**Electronic supplementary material:**

The online version of this article (doi:10.1186/s12284-017-0148-3) contains supplementary material, which is available to authorized users.

## Background

Seeds are mysterious genetic capsules of plant life cycle essential for establishment and reproduction to maintain the species from generation to generation. For the crop establishment in the field, viable seeds are crucial input. Good quality seeds of improved varieties can contribute to about 20–25% increase in productivity (Hasanuzzaman, 2015). Therefore, there is a need to sustain seed viability during storage and improve the seedling vigour. During ageing process, seeds deteriorate, lose quality, viability and vigour especially under high temperature and humidity (Kapoor *et al.,* 2011). Under increased moisture conditions, temperature and humidity, loss of seed viability is due to enzymatic and non-enzymatic modification of proteins by Amadori and Maillard reactions (Murthy and Sun, 2000; Miyata *et al.,* 2000). Seed deterioration has shown to be influenced by a number of factors related to seed structure and biochemical characteristics (Shaban, 2013). Increased lipid peroxidation leading to loss of membrane integrity, reduced energy metabolism, impairment of RNA and protein synthesis, formation of protein iso-aspartyl residues, DNA degradation, accumulation of reactive oxygen species (ROS) and reactive carbonyl compounds (RCCs) which enhances seed deterioration during storage (Mudgett and Clarke, 1993; Mudgett *et al.,* 1997; Murthy *et al.,* 2003; Kibinza *et al.,* 2006; Mohammadi *et al.,* 2011; Mano, 2012; Shaban, 2013).

Lipid peroxidation is the major cause for seed deterioration that damage integrity of cell membrane (Gidrol *et al.,* 1989). Lipoxygenases catalyze the oxidation of polyunsaturated fatty acids to produce conjugated unsaturated fatty acid hydroperoxides (Feussner and Wasternack, 2002). Lipid degradation caused by phospholipase D (PLD) activity is known to be responsible for seed deterioration in Arabidopsis (Wang *et al.,* 2012). In rice, transcript levels of *OsPLDα1* and *α3* increased during ageing. Accumulation of lipid peroxides lead to the production of reactive carbonyl compounds (RCCs) that are α, β-unsaturated aldehydes and ketones. These RCS include melondialdehyde (MDA), acrolein, 4-Hydroxy-(E)-2-nonenal (HNE) and 4-Hydroxy-(E)-2-hexenal (HHE) are most reactive and toxic that diffuse across the membrane with longer half-life and modify the proteins and nucleic acids (Sayre *et al.,* 2006; Mano, 2012). Oxidative stress is the primary cause to produce many long lived cytotoxic RCCs that form aggregates with proteins and lipids that generates advanced glycation end products (AGEs) and advanced lipoxidation end products (ALEs) which affect metabolic pathways (Ott *et al.,* 2014; Semchyshyn, 2014).

Genetic enhancement studies for improving seed viability and seedling vigour have identified several genes that are involved in regulating seed deterioration. Overexpression of *PIMT1* in Arabidopsis reduced the accumulation of L-isoaspartyl residues in seed proteome and showed increased germination and seedling vigour (Oge *et al.,* 2008). Transgenic Arabidopsis seeds overexpressing lotus (*Nelumbonucifera L*.) *NnMT2a* and *NnMT3* (metal-binding protein metallothionein) genes showed an increased resistance to accelerated ageing treatment and enhanced seedling vigour (Zhou *et al.,* 2012). DNA repair genes act during the early phase of germination and expression of *DNA ligase-I* showed improved seed vigour in Arabidopsis (Andreuzza *et al.,* 2010; Ventura *et al.,* 2012). A forward genetic approach used to validate seed longevity trait based on activation-tagging mutants lead to the identification of an *RSL1a ubiquitin ligase* that showed enhanced seed longevity (Bueso *et al.,* 2014).

During ageing, accumulation of lipid peroxidation compounds and RCC’s reduces seed viability and therefore, detoxification of these cytotoxic compounds may enhance seed viability and vigour. Aldehyde reductase from Mung bean belonging to Aldo-keto reductases (AKRs) family has been reported to detoxify a wide variety of lipid peroxidation or glycolysis derived cytotoxic compounds (Colrat *et al.,* 1999). The AKRs catalyze a variety of carbonyl compounds including aldehyde form of glucose that is reduced to corresponding sugar alcohols and sorbitol (Sree *et al.,* 2000). From this context, the focus of this study is to understand the role of cytotoxic compounds that accumulate during seed ageing and the relevance of detoxifying enzymes in seed deterioration in rice. The study demonstrates that, accumulation of RCCs during seed storage cause seed deterioration. Overexpression of RCC’s detoxifying enzyme, *Pseudomonas AKR1* (*PsAKR1*) in tobacco and rice showed improved seed viability by reducing the levels of MDA, methyl glyoxal (MG), Maillard products and Amadori compounds under controlled accelerated deterioration conditions and improved seed viability and seedling vigour.

## Results

### High temperature and humidity during seed storage affect seed viability in rice genotypes

**Fig. 1:**
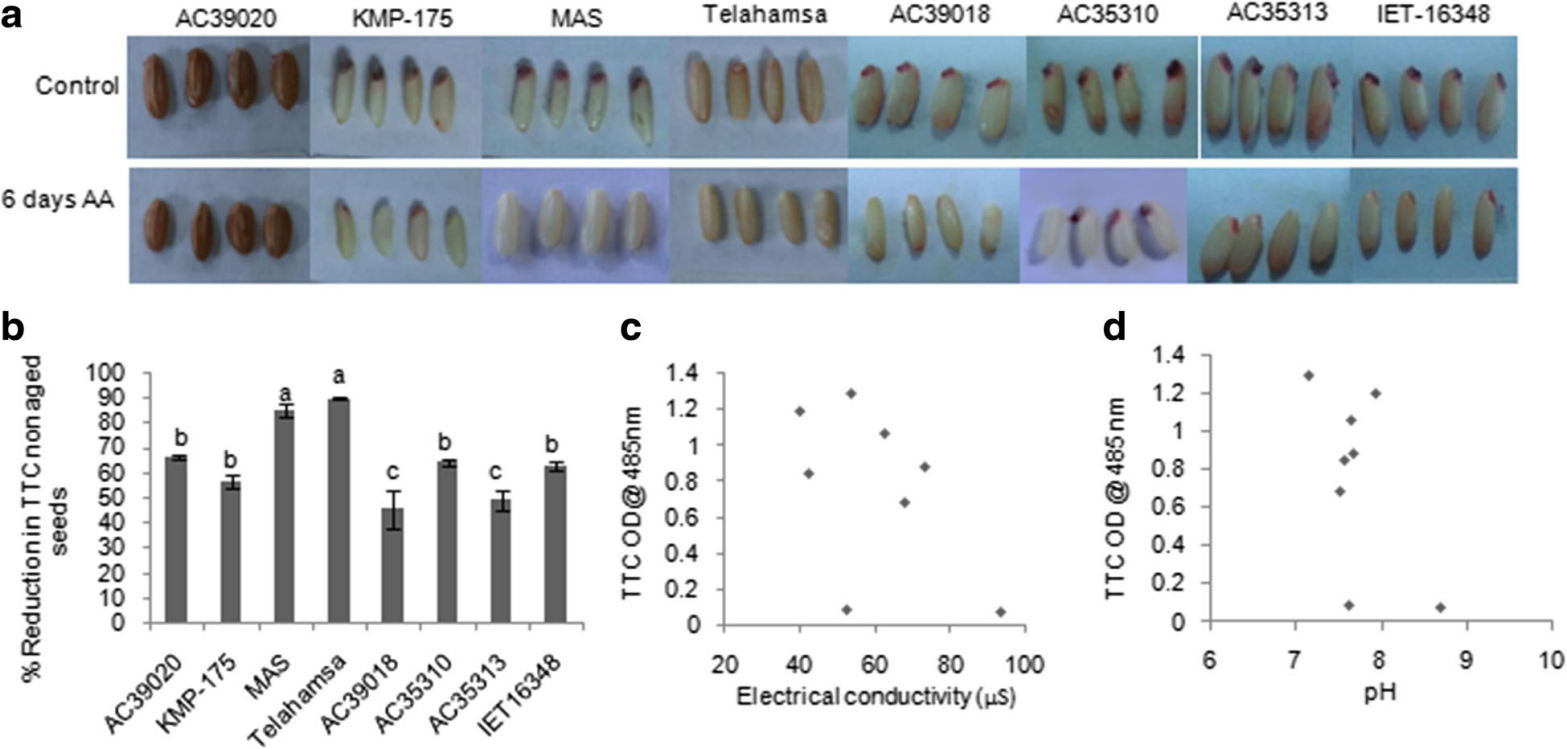
Variations in seed viability across rice genotypes under aged and non-aged conditions. **a** Photograph showing TTC staining of rice genotypes showing variation in seed viability under control and accelerated ageing conditions. The photographs were taken after one day of incubation in TTC stain. **b** Variability in dehydrogenase activity as quantified by reduction in TTC levels over control showing differences in seed viability, (*n* = 10) (Alphabets with same letters are non-significant at *p* = < 0.05). **c** Relationship between seed viability (an indication of TTC reduction) and membrane damage in rice upon ageing treatment and **d** Effect of pH on dehydrogenase activity (as indicated by TTC reduction) in rice seeds upon ageing treatment

To assess the variability in seed viability, the rice genotypes were exposed to accelerated ageing (AA) treatment for 4, 6 and 8 days at 45 °C with 100% relative humidity. All genotypes germinated after 4 days of ageing and after 8 days only two genotypes AC39020 and AC35310 showed germination. To assess differential response of the genotypes, 6 days of AA was found to be optimum and genotypes showed significant variability in seedling vigour index and germination percentage. TTC assay was carried out as a measure of dehydrogenase activity and is an indirect measure of seed viability status. TTC reduction was higher in genotypes AC39018 and AC35313 while it was less in Tellahamsa and MAS (Fig. 1a & b). Further the extent of loss of membrane damage was negatively related to seed viability. The pH of the seed leachate also showed a negative relationship with seed viability (Fig. 1c & d). The data suggests that under ageing process rice seeds undergo many biochemical changes and hence deteriorates faster and results in loss of viability.

### Accumulation of RCCs affects germination and seedling vigour in rice genotypes under accelerated ageing conditions

**Fig. 2:**
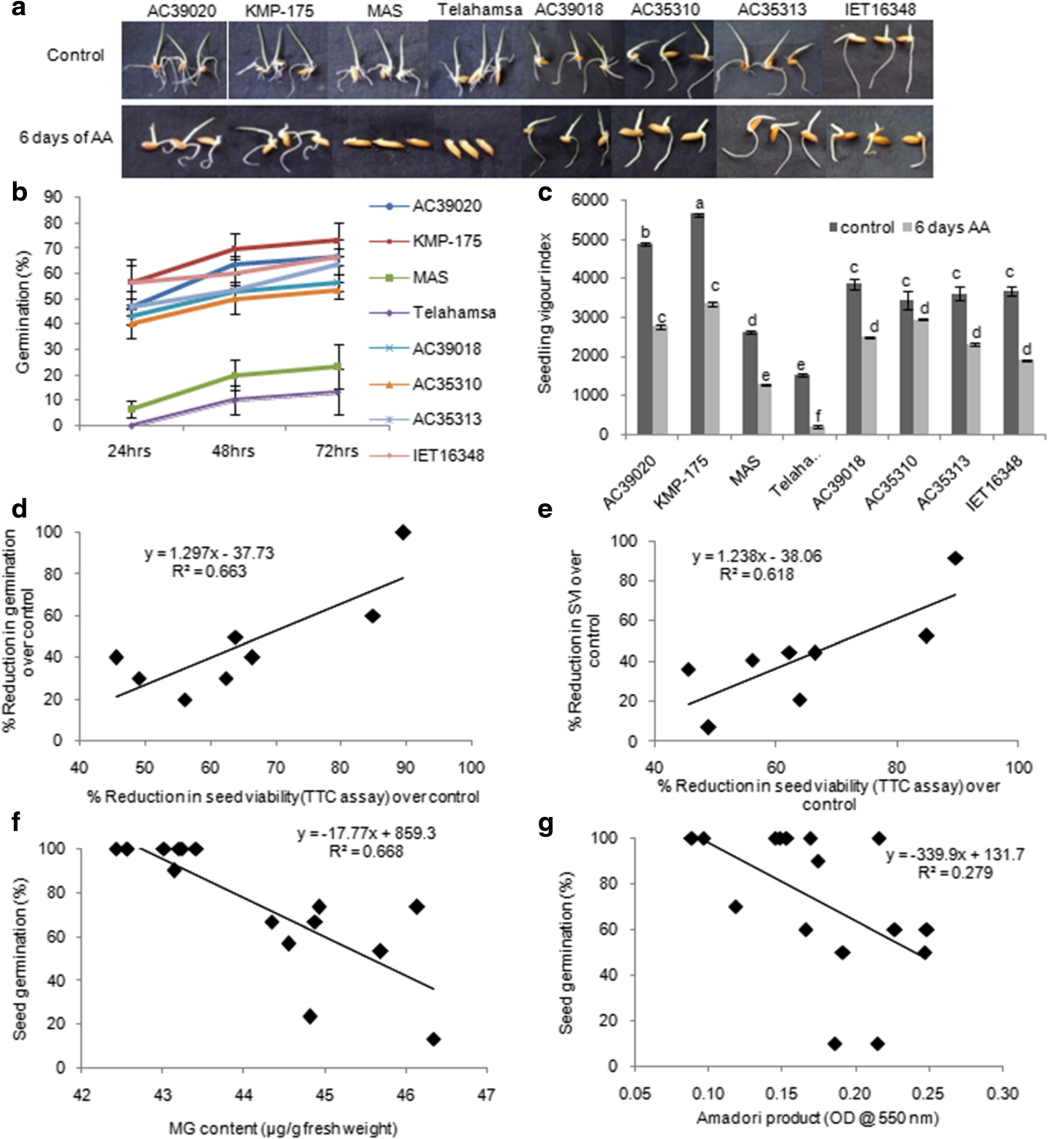
Accumulation of reactive cytotoxic compounds affects seed viability, germination and seedling vigour under ageing treatments. **a** Photograph showing germination and seedling growth of rice after accelerated ageing treatment, **b** Variability in germination with time in aged and non-aged seeds (*n* = 50). **c** Seedling vigour index (SVI) in control and aged seeds (Same Alphabets indicate non-significant at *p* = < 0.05). **d** Relationship between seed viability and seed germination and **e** Relationship between seed viability and seedling vigour index in rice, **f** Relationship between MG content and seed germination and **g** Relationship between Amadori products and seed germination

The variability in the accumulation of RCC’s and their role in seed germination were assessed in the seeds of rice genotypes exposed to accelerated ageing. Seeds subjected to accelerated ageing showed reduced germination and seedling vigour (Fig. 2a). The genotypes MAS and Tellahamsa showed less than 50% germination compared to IET16348 and KMP-175 genotypes that showed higher germination percentage (Fig. 2b). Further, the seedling vigour was also found to be significantly low in MAS and Tellahamsa. MAS, Tellahamsa and IET-16348 showed very less seedling vigour index compared to other genotypes (Fig. 2a & c). The germination speed index (GSI) an indication of time taken for seed germination was less in all the genotypes except Tellahamsa that had 8 GSI (Additional file 1: Figure S1a). The seed germination and seedling vigour showed a significant positive correlation with seed viability as measured by levels of TTC assay as a reflection of NADPH dependent dehydrogenase activity (Fig. 2d & e). Further, significantly higher accumulation of cytotoxic compounds was observed in aged seedlings. MG accumulated in seeds upon ageing treatment showed an inverse correlation seed germination (Fig. 2f). Similarly, Maillard and Amadori products, had similar effect on seed germination indicating that cytotoxic compounds affect the seed germination and seedling vigour (Fig. 2g). Amongst the genotypes, Tellahamsa and AC35313 showed higher levels of MG compared to the other genotypes (Additional file 1: Figure S1b). The increase in Maillard products was significantly higher in Tellahamsa genotype during ageing conditions (Additional file 1: Figure S1c). However, Amadori products (glycation end products) accumulated significantly higher levels in genotypes such as AC39020, AC39018 and AC35313 under ageing condition (Additional file 1: Figure S1d). Accumulation of Amadori products negatively correlated with germination percentage that affected the seedling vigour (Fig. 2g). Further, α-amylase enzyme activity was significantly increased in aged seedlings specifically in the genotypes AC39020 and KMP-175 (Additional file 1: Figure S1 e & f). These genotypes also showed higher accumulation of total sugars under ageing condition (Additional file 1: Figure S1f). The results clearly demonstrate that RCC’s accumulate during ageing and negatively influence on seed vigour.

### Overexpression of *PsAKR1* gene in tobacco detoxifies RCCs and improves seed viability and germination

**Fig. 3:**
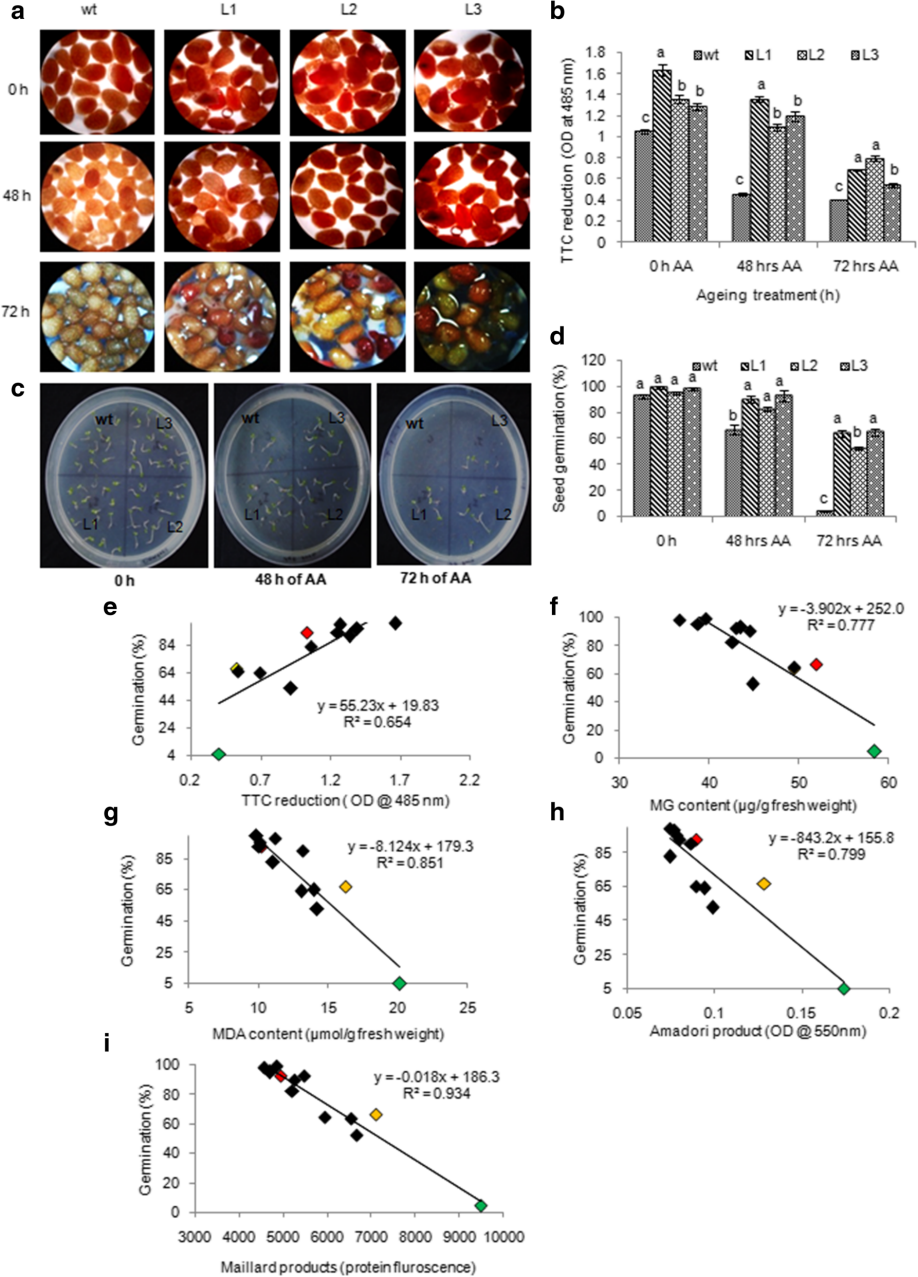
*PsAKR1* expressing tobacco transgenic seeds showed improved seed vigour and reduced reactive carbonyl compounds, **a** Transgenic seeds showing more TTC staining than wild type seeds, **b** Extent of TTC reduction (an indication of seed viability in WT and transgenic seeds at different durations of ageing treatment, **c** Differential seed germination of WT and transgenic seeds, **d** Germination percentage of transgenic and wild type seeds after accelerated ageing treatments, error bar indicate mean values from three biological replicates that are having 10 seeds each. *t*-test for both wildtype and transgenic seeds were conducted. Alphabets with same letters are non-significant at *p* = < 0.05. **e**, **f** **g**, **h** and **i** Correlations between germination % with TTC, methylgloxal (MG) content, malondialdehyde (MDA) content, Amadori product and Maillard products. Data points in colour indicate different time points of wild type seeds exposed to aging conditions at control (*red*), 48 h (*yellow*) and 72 h (*green*)

In rice genotypes, reduced seed viability and germination was correlated with increased accumulation of RCCs suggesting that managing these compounds is crucial to improve seed viability, germination and vigour. In view of this, to reduce the accumulation of RCC’s in seeds one of the RCC detoxifying enzymes, Aldo-ketoreductase-1 from *Pseudomonas* strain (PsAKR1) that was codon optimized to plants and overexpressed in tobacco. The stable differentially expressed transgenics lines (Additional file 1: Figure S2a) of T3 generation were used to study the seed viability and vigour by exposing to accelerated ageing treatment for 48 and 72 h. The transgenic seeds after accelerated ageing showed higher levels of TTC reduction suggesting sustained NADPH dependent dehydrogenases activity compared to wild type seeds (Fig. 3a & b). Under normal conditions, transgenic seeds showed early germination than the wild type seeds. After 48 h of ageing treatment, transgenic seeds showed ~80% germination whereas, wild type seeds showed 65% germination. Even after 72 h of ageing transgenic seeds showed more than 50% seed germination whereas wild type seeds showed less than 10% germination (Fig. 3c & d). The germination percent of transgenic seeds was positively correlated with higher dehydrogenase activity (Fig. 3e). Further, the accumulation of MDA and MG was less in transgenic seeds compared to wild type seeds (Additional file 1: Figure S2b & c) and the levels of these compounds negatively associated with the seed germination (Fig. 3f & g). Even the accumulation of Amadori and Maillard products under ageing conditions was significantly low in transgenic plants (Additional file 1: Figure S2d & e). The low level of RCCs including AGEs in tobacco transgenics indicates efficient detoxification of RCCs by *PsAKR1* (Fig. 3).

### Less viable and susceptible rice genotype (Tellahamsa) under ageing condition was rescued by overexpression of *PsAKR1*

**Fig. 4:**
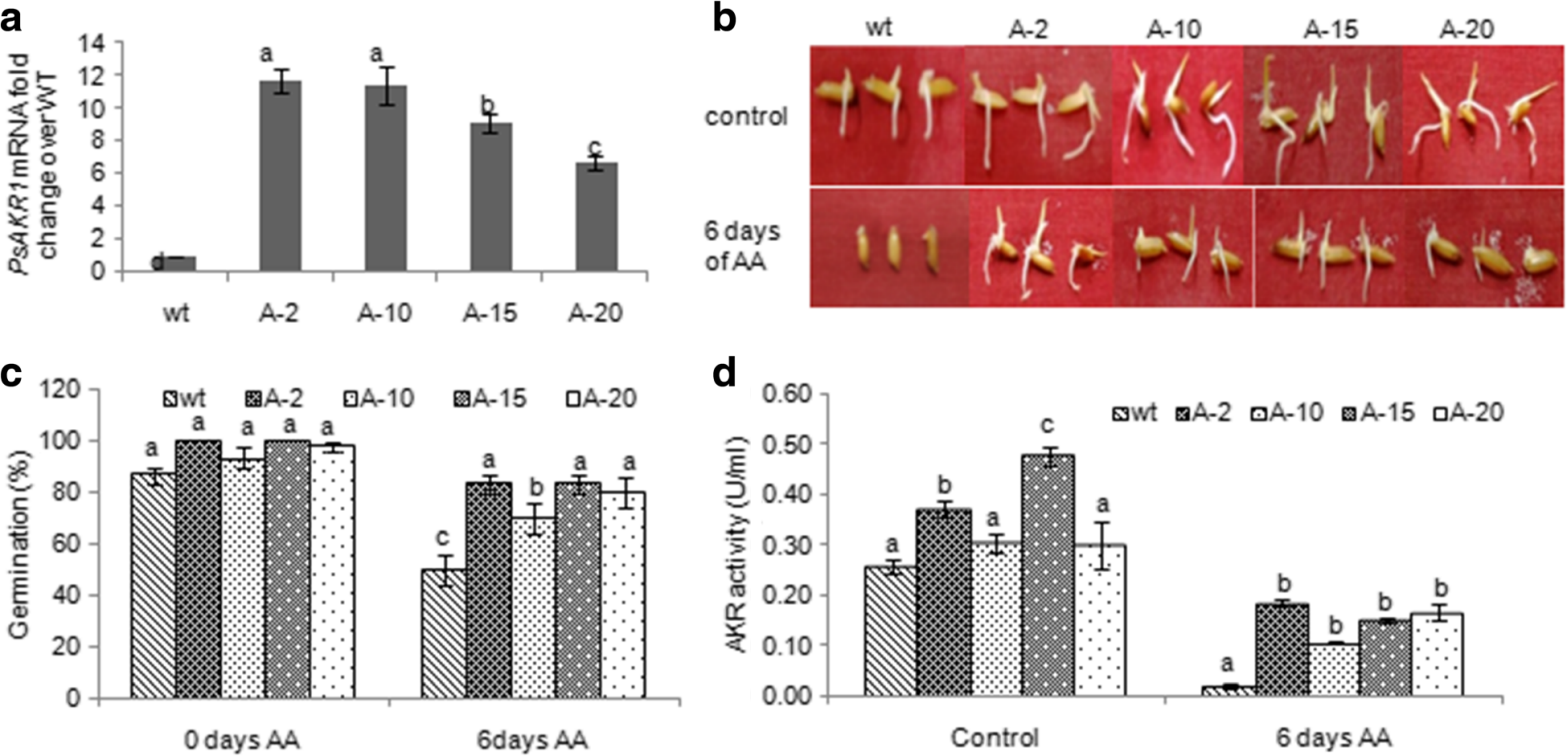
Overexpression of *PsAKR1* in susceptible rice genotype Tellahamsa showed improved seed viability and germination. **a** Transcript levels of *PsAKR1* in different transgenic rice lines, The RNA from seeds were isolated and the cDNA is used as a template, **b** Photograph of transgenic rice seeds showing early germination after ageing treatment, **c** Germination percentage of transgenic seeds under 0 day and 6 days of AA is more compared to wild type Tellahamsa seeds, **d** AKR1 enzyme activity in rice seeds under control and ageing conditions. The total protein was extracted and enzyme activity was determined against Methylglyoxal as substrate. Minimum of three biological replicates were used in all the assays

**Fig. 5:**
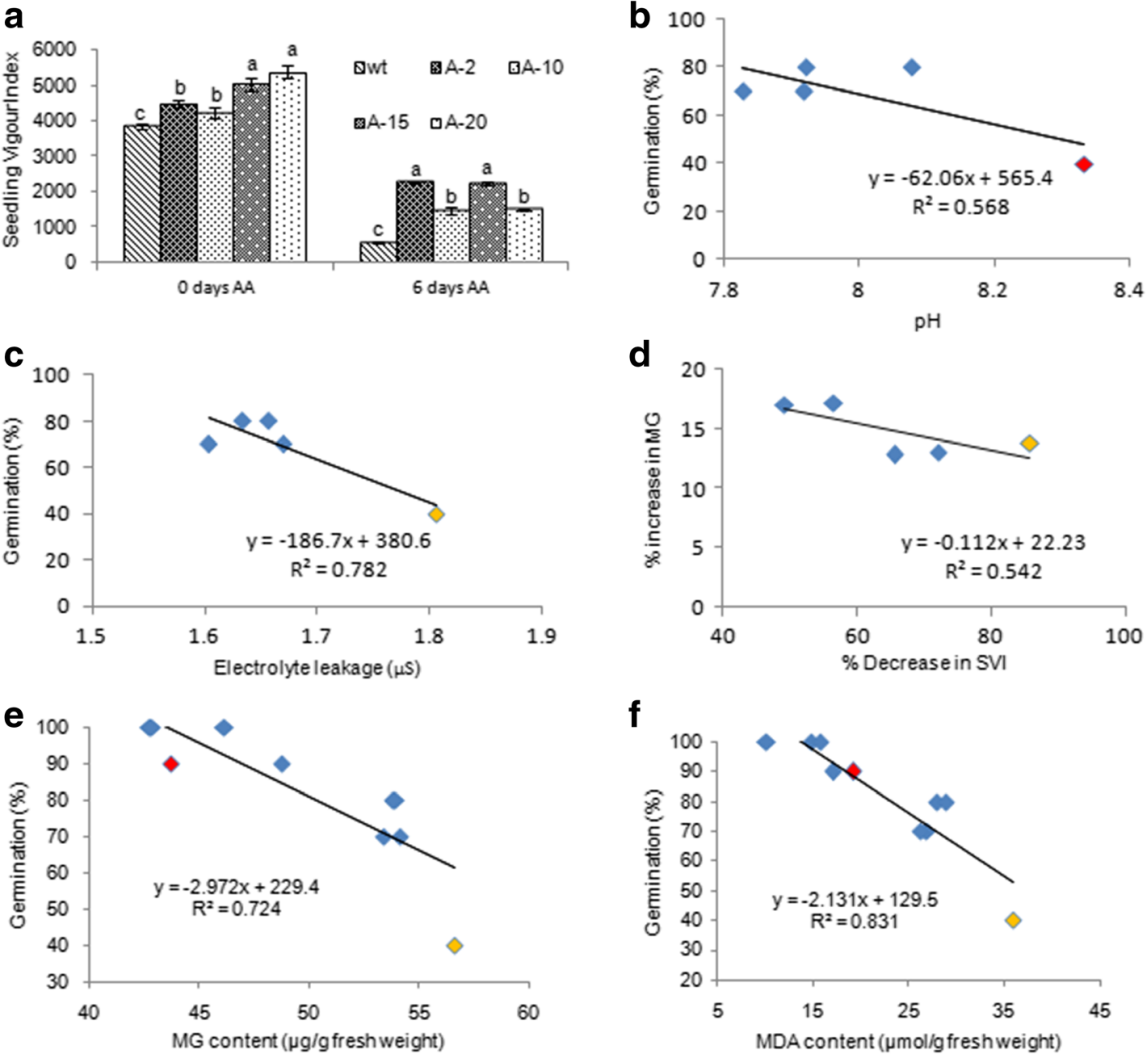
Overexpression of *PsAKR1* in Tellahamsa genotype rescued seed viability by scavenging cytotoxic compounds. **a** Transgenic seeds showed higher seedling vigour index compared to wild type Tellahamsa seeds, different letters above the error bars indicate significance levels, *n* = 10, three biological replications with minimum 10 seeds were tested for ageing treatments. Student *t*-test for both wild type and transgenic seeds were conducted. Alphabets with same letters are non-significant at *p* = < 0.05. **b** and **c** Correlation between germination percentage with electrolyte conductivity (EC) and pH of the seed leachate of rice transgenic and wild type seeds after soaking in water. **d** Correlation between reduction in methylglyoxal (MG) with seedling vigour index (SVI). **e** and **f** Correlation between germination with MG content and malondialdehyde (MDA) content. Data points in different colours indicate wild type seeds exposed to control (*red*), 48 h (*yellow*) and 72 h (*green*) of aging conditions

**Fig. 6:**
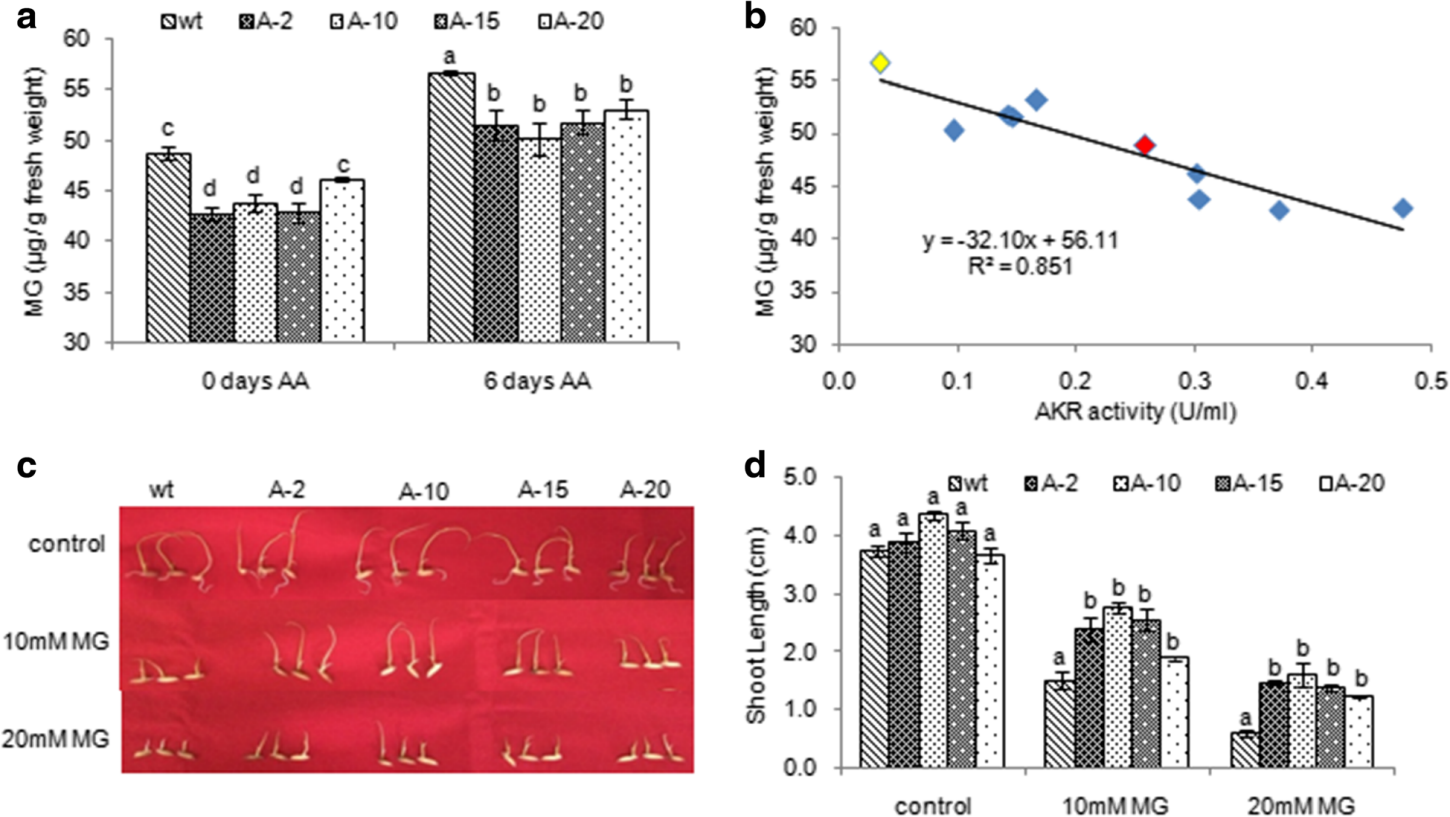
The rice transgenics expressing *PsAKR1* gene showed reduced methylgloxal (MG) levels in seeds under ageing conditions. **a** MG levels in transgenics and wild type seedlings under control and 6 days of accelerated ageing, **b** Correlation between reduction in MG with increased AKR activity, **c** Response of wild type and transgenic seedlings to 10 mM and 20 mM MG after 4 days of treatment, **d** Shoot length of wild type and transgenics seedlings on MG treatment, *n* = 10. Red and yellow dots represent values for wild type in control and ageing conditions respectively

To improve the seed viability of susceptible rice genotype Tellahamsa, transgenic plants expressing AKR1 were developed and stable lines were identified by screening on glyphosate as described in our previous study (Vemanna et al., 2016). The transgenic plants were confirmed for presence of transgene using genomic DNA PCR (Additional file 1: Figure S3a). Further based on the expression profile four transgenic lines with differential transcript levels were selected to assess the seed viability and seedling vigour (Fig. 4a). Transgenic rice seeds upon ageing treatment showed higher seed viability that was reflected in improved seed germination compared to wild type seeds. The transgenic seeds showed higher per cent germination than the wild type seeds under normal as well as ageing conditions (Fig. 4b & c). Further the AKR1 activity in transgenic seeds was significantly higher compared to wild type seeds (Fig. 4d). Under ageing conditions the AKR1 activity was reduced even in transgenics, however the transgenic seeds still maintained adequate activity to maintain the seed viability (Fig. 4d). The seedling vigour index was also higher in all the transgenics compared to wild type plants (Fig. 5a). Further, the transgenic seeds had significantly less electrolyte leakage than wild type seeds (Additional file 1: Figure S3b). The pH of the seed leachate was less acidic in transgenics whereas wild type seed leachate had alkaline pH (Additional file 1: Figure S3c). The EC and pH were negatively correlated with seed germination and seedling vigour index (Fig. 5b & c). Similarly the levels of MG is negatively influenced on seedling vigour index (Fig. 5d). The transgenic seeds under ageing treatment showed significantly reduced levels of MG and MDA (Fig. 6a & Additional file 1: Figure S3d) and therefore, a significant negative relationship was observed between cytotoxic compounds and seed germination (Fig. 5e & f).

### The AKR1 transgenic seeds detoxify the cytotoxic MG to improve seed viability and vigour

The transgenic rice seeds expressing AKR1 showed reduced MG levels and the increasing activity in the transgenic lines corelates with the reduced MG levles both in controlled and ageing treatments (Fig. 6b). Further, to assess whether overexpression of AKR1 in susceptible rice genotype Tellahamsa rescues the seedlings from MG induced effect, the growth response of germinated seedlings was assessed at 10 mM and 20 mM MG. In both the concentrations of MG, the transgenic seedlings had maintained higher growth and shoot growth was less affected compared to wild type seedlings (Fig. 6c & d). Higher AKR activity in transgenic seeds detoxify the MG and enhances the seed viability and vigour. These results demonstrated that, detoxifying the RCCs that are accumulated during ageing conditions by AKR1, improve seed viability and seedling vigour. Therefore overexpression of *PsAKR1*in Tellahamsa genotype rescued the susceptible phenotype during ageing process.

## Discussion

Seed viability and seedling vigour has phenomenal relevance in crop establishment and productivity especially under abiotic stress conditions. However, during storage, seed viability reduces rapidly with the accumulation of many cytotoxic compounds. Seeds deteriorate because of high temperature and relative humidity (RH) leading to oxidative damage to lipids, proteins and nucleic acids affecting cellular metabolic activities (Mano, 2012). Oxidative damage specifically on lipids produces peroxidation compounds like MDA and MG that are highly reactive electrophiles. These compounds further acts on proteins through Maillard reaction or Schiff’s bases to form Amadori products that leads to the production of AGE’s and ALE’s (Miyata *et al.,* 2000). From this context, managing RCC’s have greater relevance in improving the seed viability. It was reported that, different eco-geographic regions are responsible for the variation in japonica cultivars having low longevity than upland cultivars (Rao and Jackson 1996). The genotypic variation in indica rice genotypes that are tested in this study showed variability in seed viability under ageing conditions. The accumulation of reactive carbonyl compounds in rice genotypes exposed to seed ageing showed genotypic variability and it was related to seed germination. The rice genotypes Tellahamsa and MAS showed early loss of seed viability and germination mainly due to accumulation of cytotoxic compounds. The reduced dehydrogenase activity was tightly linked with seed viability. The susceptible and less viable genotypes such as Tellahamsa and MAS showed distinctly low dehydrogenase activity and membrane stability compared to rest of the genotypes. The accumulation of Maillard and Amadori products signifies these two genotypes accumulated more AGEs and ALEs under ageing conditions. The Maillard reaction product has been shown to impair protein function leading to browning reaction and thus affecting the metabolic activity (Wettlaufer and Leopold 1991). The results from genetic variability in studies suggest that, RCC accumulation significantly affects the seed viability in rice.

The enhanced level of α-amylase activity in all the rice genotypes when exposed to higher temperature during ageing treatment was evident with the accumulation of higher levels of total sugars. However, a significant variation in the genotypes was observed. Increased rate of germination at higher temperature is mainly attributed to enhanced respiration with the accumulation of reducing sugars. The α-amylase activity during germination is considered as a biomarker in several species (Koning, 1994). In this study, the increased α-amylase activity resulted in accumulation of more sugars in rice genotypes under ageing conditions. However, in spite of higher α-amylase activity, rice genotypes did not show increased germination. Instead, our results showed that the increased sugar content in Tellahamsa and MAS was negatively correlated with germination percentage. The increased α-amylase activity during ageing leads to accumulation of sugars and their utilization in respiratory metabolism largely depends on the activity of dehydrogenases associated with citric acid cycle. During AA, the dehydrogenase activity was reduced, that may have resulted in the conversion of glucose into MG. During photosynthesis CO_2_ converts in to sugars, and during respiration auto oxidation of sugars is inevitable and produces ROS and sugar derived RCCs (Takagi *et al.,* 2014). In microbes the enzymatic conversion of dihydroxyacetone phosphate catalysed by MG synthase produces MG (Richard, 1984). The MG, Maillard and Amadori products thus produced during AA conditions affected the cellular enzymes thus affecting the metabolic activity. The higher level of RCC’s under ageing conditions observed in this study seems to be the major contributing factor for loss of seed viability and vigour in rice genotypes. An inverse relationship between cytotoxic compounds and seed viability was observed in several crop species (Murthy *et al.,* 2003). In soybean, increase in Maillard reaction during accelerated ageing caused enhanced seed deterioration (Sun and Leopold 1995).

Managing the levels of RCCs is crucial to sustain the seed viability. From this context, RCC detoxifying enzymes assumes greater significance. The null rice mutants of Aldehyde dehydrogenase (*ALDH7*) showed increased MDA resulting in reduced seed viability (Shin and Kim 2009). The importance of AKR’s in scavenging RCC has been shown in several other studies related to stress response. The reduced RCCs in rice, Arabidopsis and Alfalfa by overexpression of Aldo-keto reductase family genes showed increased tolerance to salinity, drought and heavy metals. The *E. coli* expressing *OsAKR1* showed abiotic stress tolerance by detoxifying MG (Turoczy *et al.,* 2011). Constitutive expression of *Medicago sativa ALR1* in tobacco plants imparted tolerance to hydrogen peroxide, paraquat, salt and dehydration stresses (Oberschall, *et al.,* 2000; Bartels, 2001). The detoxification of reactive carbonyl glyphosate by overexpression of *PsAKR1* and *OsAKR1* in tobacco improved tolerance against herbicide (Vemanna *et al.,* 2016). The overexpression of *PsAKR1* in tobacco showed enhanced protection of cellular enzymes from the salinity induced RCCs (Vemanna *et al*., 2017). From this context, increased seed longevity could be associated with the detoxification of carbonyl compounds that are substrates for Aldo-keto reductases. The transgenic tobacco and rice seeds expressing *PsAKR1* showed reduced accumulation of RCCs like MDA, MG, Maillard products and Amadori compounds. The AKR activity in transgenics is correlated with the levels of MG and enhanced shoot growth of transgenics on MG further signifies that AKRs potentially detoxify these cytotoxic compounds. This resulted in reduced seed deterioration and enhanced viability of seeds when exposed to ageing treatment. The reduced levels of lipid peroxidation derived aldehydes found in the transgenic lines of tobacco and rice signifies the potential of *AKR1*. This shows that *AKR1* expressing transgenic plants had improved detoxification ability of RCCs.

## Conclusion

In this study, the relevance of reactive cytotoxic compounds was assessed in rice genotypes by exposing to accelerated aging conditions. Under aging treatment the reactive cytotoxic compounds accumulated in all the genotypes and affect the seed viability. The genotypic variability is correlated with the accumulation of MDA, MG and Maillard products. Further, we characterize an Aldo-keto reductase (AKR) by developing transgenics in tobacco and rice susceptible genotype Telahamsa. The transgenics seeds showed improved AKR activity, viability and germination when exposed to the ageing treatments. The reduced levels of RCCs MDA, MG, Maillard and Amodari compounds in transgenics signifies that RCCs are detoxified by the AKRs. The recovery in seed viability in highly susceptible rice genotype Tellahamsa by overexpression of *AKR1* clearly suggests that, detoxification of cytotoxic compounds by Aldo-keto reductases play a crucial role in maintaining the seed longevity and seedling vigour. The study demonstrates that, efficient detoxification of RCC’s by overexpression of *AKR1* enhances the life span of seeds.

## Methods

### Plant material

Freshly harvested seeds of rice genotypes AC39020, KMP-175, MAS, Tellahamsa, AC39018, AC35310, AC35313 and IET-16348 with known physiological and stress tolerant traits were used in the present study. To develop transgenic plants overexpressing *PsAKR1* driven by ribulose 1,5-bis phosphate carboxylase (RBCS) promoter in *pBINplus* binary vector, rice genotype Tellahamsa and tobacco variety KST-19 were used. The Agrobacterium mediated in-planta transformation protocol for rice (Babitha 2012) and tissue culture based transformation protocol for tobacco was followed to develop transgenic plants. For rice inplanta transformation pre-germinated seed embryonic axis was wounded with a fine needle and the *PsAKR1* construct harboring agrobacterium was infected. Since AKRs detoxify glyphosate, the promising rice transgenics were identified by screening on glyphosate in quartz sand as described in our previous study (Vemanna et al., 2016). The homozygous T2 generation seeds were used to assess the seed viability and vigour. Total genomic DNA was isolated from seedlings of wild type and transformed plants following the CTAB method (Dellaporta et al., 1983). PCR analysis was performed to amplify *AKR1* gene using the primers (For primers, please see Additional file 1: Table S1) and the products were electrophoresed on a 1% agarose gel.

The total RNA was isolated according to Datta et al., (1989) to confirm the *PsAKR1* expression in transgenic tobacco and rice seeds. The cDNA was synthesized by oligo (dT) primers using Moloney murine leukemia virus reverse transcriptase (MMLV-RT; MBI Fermentas, Bangalore). The qRT-PCR analysis was done using cDNA pool as a template. The gene specific primers were used to confirm the expression levels by qRT-PCR. The relative expression level of *AKR1* was calculated using the Image J software based on the band intensity. Actin was used as internal control for normalization (for primer, please see Additional file 1: Table S1).

### Accelerated ageing/controlled deterioration of seeds

Dry seeds of rice genotypes were subjected to a standardized accelerated ageing treatment of 45 °C with 100% relative humidity for 6 days. Similarly, transgenic rice and tobacco seeds overexpressing *PsAKR1* were also subjected to ageing treatment for 6 days and 3 days respectively. Seeds in paper covers were placed inside desiccators with water to maintain 100% RH and were kept inside incubator (Delouche and Baskin 1973). After 3 and 6 days of incubation, seeds were removed from the desiccators and subjected to germination assay.

### Methyl glyoxal treatment assay

Transgenic and wild type seeds were pre-germinated and placed on agar plates with half MS media supplemented with 10 mM and 20 mM MG. After 4 days the shoot length of the seedlings was recorded in both the treatments.

### Germination and seedling growth

The seeds removed from the accelerated ageing treatment and from control conditions were imbibed for 4 h and then placed in petri plates with moistened blotting paper. Ten seeds from each genotype were used for the study with three replications. After two days, the percent seed germination was measured and later, root length and shoot length was also measured. Based on the data generated, germination speed index (GSI) was calculated (AOSA, 1983) with some modifications. GSI = (Total no. of seeds sown/initial germinated seeds) + (Total no. of seeds sown/ final germinated seeds). In addition to GSI, seedling vigour index was also calculated using the formula, SVI = Germination percentage x (root length + shoot length) (Abdul-Baki and Anderson, 1973).

### pH of the seed leachate media

Both accelerated aged and control seeds were soaked in a known volume of water overnight and the pH of the seed leachate was measured using digital pH meter (Systronics, μpH system 361, Delhi, India).

### Electrolyte leakage in the media

After accelerated ageing treatment, the seeds were soaked in a known volume of water overnight and the electrolyte leakage (EC) from seed leachate was measured using electrical conductivity meter (Elico, CM180, Hyderabad, India) and expressed in micro Siemens (μS). Initial EC was measured in water before soaking the seeds and then seeds were soaked in water overnight and final EC was measured from the leachate. The electrolyte leakage was calculated using the following formula: EC (%) = (initial EC/final EC) × 100.

### Assessment of RCC’s upon ageing treatment

Reactive carbonyl compounds like MDA, MG, Amadori and Maillard products were quantified in rice genotypes as well as in tobacco and rice transgenics overexpressing PsAKR1 gene.

### MDA

About 0.5–1.0 gram of seeds exposed to ageing treatment was homogenized in 5 ml of 10% (W/V) trichloroacetic acid (HiMedia, Nasik, Maharashtra) and 0.25% of thiobarbutiric acid. The homogenate was centrifuged at 12,000 rpm for 15 min at room temperature. The supernatant was mixed with an equal amount of thiobarbutiric acid [0.5% in 20% (W/V) trichloroacetic acid] (Sigma aldrich, Bangalore, India) and the mixture was boiled for 25 min at 100 °C followed by centrifugation for 5 min at 7,500 rpm to clarify the solution. Absorbance of the supernatant was measured at 532 nm and 600 nm and corrected for nonspecific turbidity by subtracting the absorbance at A600. The standard MDA (Sigma Aldrich, Bangalore, India) was used to develop the standard graph.

### Methyl glyoxal (MG)

MG was quantified in aged and control seeds according to Yadav et al., (2005). One hundred mg of tissue was taken and ground in a known volume of distilled water and centrifuged at 11,000 rpm for 10 min at 40C and supernatant was collected. To quantify the MG content, 250 μl of 7.2 mM of 1,2-diamino benzene (1,2-phenylenediamine), 100 μl of 5 M perchloric acid and 650 μl of the neutralized supernatant were added. The absorbance was read at 336 nm using spectrophotometer (Spectra max plus-384, Spinco Biotech pvt ltd, Bangalore).

### Amadori and Maillard products

One hundred mg seeds of both control and aged seeds were ground in 1.2 ml of 50 mM phosphate buffer (pH 7.2). The homogenate was vortexed and centrifuged at 12,000 rpm for 15 min. Further, ammonium sulphate of 0.5 g ml-1 was added to precipitate the proteins. The pellet was dissolved in 3.3 ml phosphate buffer (50 mM, pH 7.2). Extracted proteins were used to measure the Amadori reaction products and Maillard reaction products. The Amadori reaction products were measured using the nitro-blue tetrazolium (NBT) method (Wettlaufer and Leopold, 1991). To this, 1 ml of NBT reagent (0.5 mM NBT in 100 mM sodium carbonate, pH 10.3) was added to 0.2 mg of extracted proteins and incubated at 400C in a water bath. The absorbance at 550 nm was recorded after 10 and 20 min of incubation using spectrophotometer.

The content of Maillard reaction products was determined using the fluorescence method using flurometer (Fluromax, Jobinyvonhoriba, USA). Extracted seed proteins (0.3 mg ml-1) were scanned with an excitation wavelength from 270–400 nm and emission wavelengths from 320–500 nm. A new fluorescence maximum was detected at the excitation of 350 nm and emission of 420 nm (Murthy and Sun, 2000). The intensity of the new fluorescence peak was used to express the content of Maillard reaction products in seed proteins. Proteins were quantified and BSA was used to develop standard graph.

### Alpha amylase activity

Amylase activity was determined by DNS (Di-nitro-salicyclic acid) method (Bernfeld, 1955). Glucose solution of 400 μg/ml and 1% starch solution was used as standard, One gram seed from both control and aged treatment was weighed and incubated in water for a known period of time and later homogenized with 9 ml of phosphate buffer (pH-7.5). The homogenate was centrifuged at 3,000 rpm for 10 min. To measure the enzyme activity 1.5 ml of 0.1 M phosphate buffer (pH-7.5) was added and the mixture was incubated at room temperature for 5 min and then added the enzyme mix. The reaction was allowed to continue for 20 min and was stopped by the addition of 2 ml DNS reagent. The test tubes were placed in a boiling water bath for 10 min and cooled. The optical density of the orange yellow coloured complex was measured at 540 nm and the values were recorded. The extent of reducing sugar formed (an indication of starch hydrolysis due to the action of amylase activity) was determined by reading the OD values against glucose standard graph.

### Total sugar content estimation

Anthrone method was followed to quantify the total sugars. One gram seed tissue from both control and aged treatment was immersed separately in ethanol and allowed to boil for 10 min. The tissue was ground and filtered until complete removal of alcohol soluble substances. The extracts were then centrifuged at 8000 rpm and later made up the volume to 10 ml with distilled water. One ml of aliquot was pipette out and made up to the volume to 2.5 ml with distilled water and added 5 ml of Anthrone reagent slowly. Then the reaction mixture was heated on boiling water bath exactly for 7.5 min and cooled immediately in ice bath. After cooling, the absorbance of the solutions at 630 nm was measured. The sugar content was calculated through standard glucose curve (Loewus, 1952).

### Measurement of TTC activity

Hulled seeds of both control and aged treatment were pre-conditioned by soaking in distilled water at 28 °C for 4 h and transferred them in 1% tetrazolium chloride solution for 6 h at room temperature in dark, and then washed several times with distilled water to remove excess solution. Two hundred mg of seeds incubated in TTC solution was ground in 1 ml of SDS and centrifuged at 8,000 rpm for 20 min. Later, the supernatant was collected and the extent of colour development was assessed based on OD values at 485 nm in spectrophotometer.

### Measurement of AKR activity

The AKR activity in the seeds was measured using MG as substrate. The total protein from 0.4 g of seeds was extracted in 400 μl of isolation buffer [25 mM Tris– HCl pH 7.6, 15 mM MgCl2 15 mM ethylene glycol tetraacetic acid, 75 mM NaCl, 60 mM b-glycerophosphate, 2 mM 1,4-DithioL-threitol, 0.1% Igepal CA-630 (Sigma–Aldrich), 1 mM NaF, 1 mM phenylmethanesulfonylfluoride (Sigma–Aldrich)]. The homogenate was centrifuged for 10 min at 13,000 g and the supernatant was saturated using 25% ammonium sulphate. The proteins precipitated were dissolved in small amount of 0.1 M sodium phosphate buffer, pH 7. All steps were done at 0–4 °C. Total protein content is quantified using Bradford’s method. The AKR activity was determined using 1 μg of crude protein and 0.1 mM NADPH and 2 mM Methyl glyoxal substrate (Sigma–Aldrich). The absorbance at 340 nm at 25 °C to monitor decrease in NADPH was quantified using spectrophotometer (Spectra max plus-384, Spinco Biotech pvt ltd, Bangalore) and AKR activity was calculated using the molar extinction co-efficient (0.623) (Turcozy et al., 2011).

## Additional file

Additional file 1: Table S1. List of Primers used in the study. **Figure S1.** Differential accumulation of RCC’s affected germination after ageing treatments in rice genotypes determined genotypic variation. **Figure S2.** Accumulation of different reactive carbonyl compounds in transgenics tobacco seeds expressing *PsAKR1.* **Figure S3.** Overexpression of *PsAKR1* in susceptible genotype Tellahamsa showed reduced malondialdehyde and less electrolyte leakage. (PDF 1708 kb)
