# Erratum to: *Aldo-ketoreductase 1* (*AKR1*) improves seed longevity in tobacco and rice by detoxifying reactive cytotoxic compounds generated during ageing

**DOI:** 10.1186/s12284-017-0160-7

**Published:** 2017-05-12

**Authors:** Kodadinne Narayana Nisarga, Ramu S. Vemanna, Babitha Kodekallu Chandrashekar, Hanumantha Rao, Amaranatha Reddy Vennapusa, Ashwini Narasimaha, Udayakumar Makarla, Mohan Raju Basavaiah

**Affiliations:** Department of Crop Physiology, University of Agriculture Sciences, GKVK, Bengaluru, 560065 India

## Erratum

After publication of the original article [1] it was brought to our attention that author Kodadinne Narayana Nisarga was incorrectly included as Nisarga Kodadinne Narayana. The correct name is included in the author list of this erratum and updated in the original article.
