# Supplementary material for: Aldo-ketoreductase 1 (AKR1) improves seed longevity in tobacco and rice by detoxifying reactive cytotoxic compounds generated during ageing
Source: Rice (N Y). 2017 Apr 13;10:11. doi: 10.1186/s12284-017-0148-3 (PMC5391344; doi:10.1186/s12284-017-0148-3)
Supplement: Additional file 1: Table S1. — List of Primers used in the study. Figure S1. Differential accumulation of RCC’s affected germination after ageing treatments in rice genotypes determined genotypic variation. Figure S2. Accumulation of different reactive carbonyl compounds in transgenics tobacco seeds expressing PsAKR1. Figure S3. Overexpression of PsAKR1 in susceptible genotype Tellahamsa showed reduced malondialdehyde and less electrolyte leakage. (PDF 1708 kb) [file 12284_2017_148_MOESM1_ESM.pdf]

Supplementary Table S1: List of primers used in the study.

Primers	Sequence
AKR1F	GATGCACAGAGAGGACGATAGC
AKR1R	ATCAAAGCTGCGCCTTGTAGC
RTAKR1F	CCGGCAACCCTCACATCAAAAGATTGGC
RTAKR1R	CGAGGCATTGGAGGCAGGTCACAGATTCC
ActinF	TCCATAATGAAGTGTGATGT
ActinR	GGACCTGACTCGTCATACTC

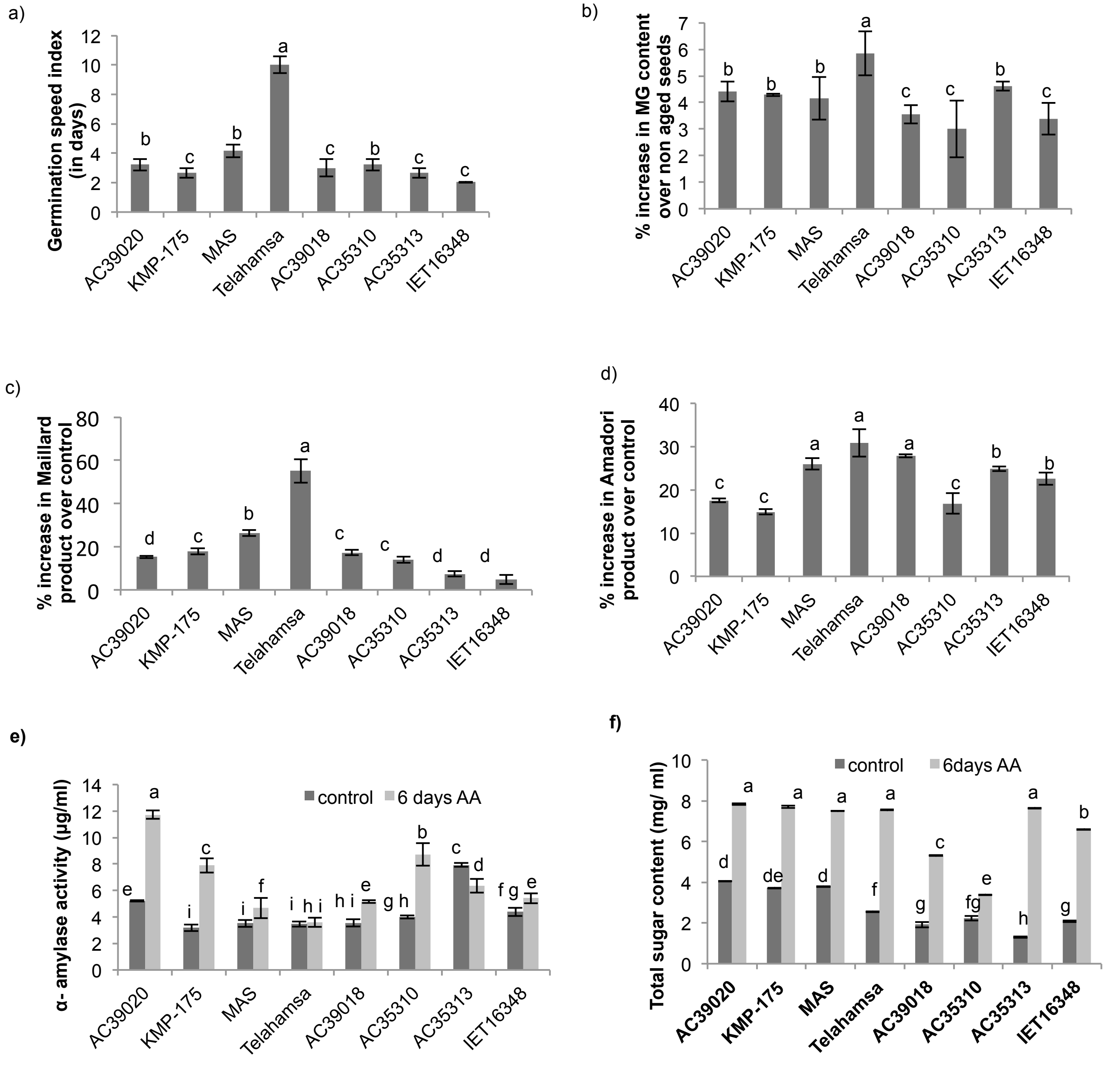

Supplementary Figure S1: Differential accumulation of RCC's affected germination after ageing treatments in rice genotypes determined genotypic variation, a) Germination speed index; time taken for germination after ageing treatments. b) Methyl glyoxal (MG) content, c) Maillard product, d) Amadori product levels, e) Reducing sugar level (an indication of starch hydrolysis due to α- amylase activity) and f) Total sugar content in rice seeds exposed to control and 6 days of ageing conditions.

Experiments were repeated minimum three times with three biological replications. Different letters above the error bars indicate significance levels, n=50, Two way ANNOVA at $P < 0.05$ between genotypes were tested. .

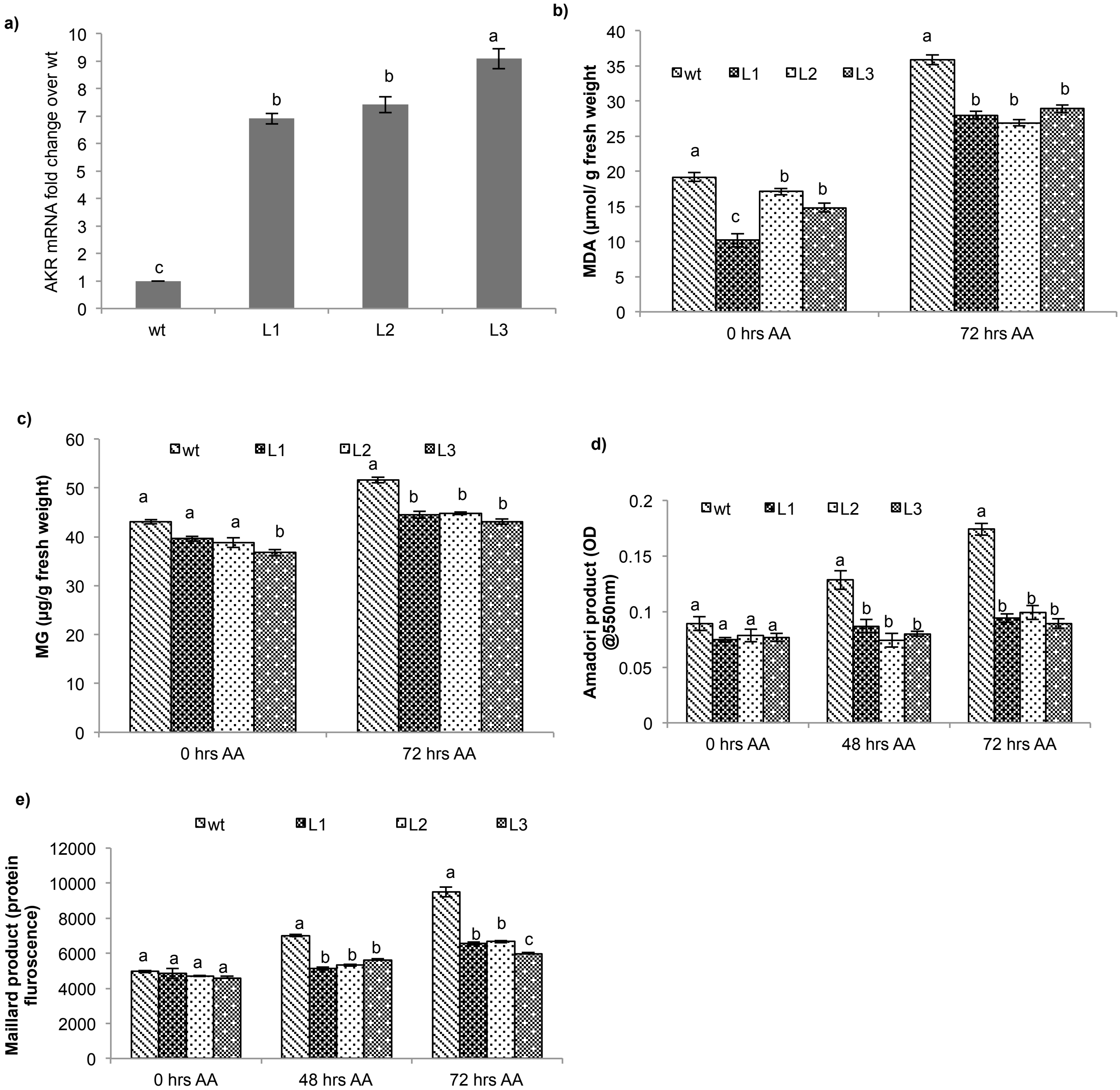

Supplementary Figure S2: Accumulation of different reactive carbonyl compounds in transgenics tobacco seeds expressing *PsAKR1,* a*)* Transcript levels of *PsAKR1* in transgenic tobacco seeds*,* b) Malondialdehyde (MDA) levels, c) Methyl glyoxal (MG) levels, d) Amadori products and e) Maillard products in transgenic and wild type seeds exposed to accelerated ageing treatments. Different letters above the error bars indicate significance levels, Student t-test at $P < 0.05$ between wild type and transgenic seeds were tested. Three biological replications were maintained for experiments with a minimum 10 seeds for biochemical compound quantification. Experiments were repeated three times.

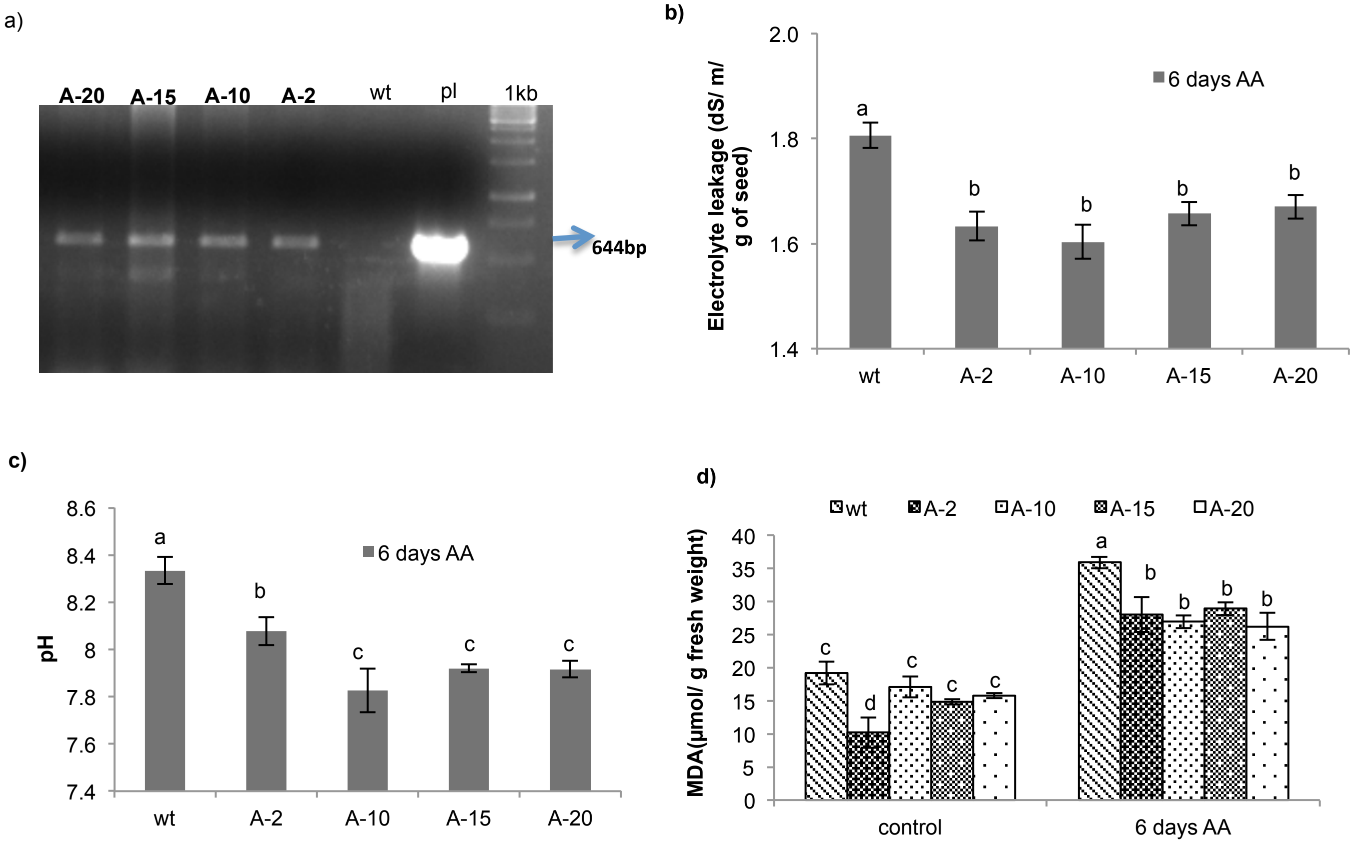

Supplementary Figure S3: Overexpression of *PsAKR1* in susceptible genotype Tellahamsa showed reduced malondialdehyde and less electrolyte leakage. a) Molecular confirmation using genomic DNA PCR, b) Electrolyte leakage (EC) and c) pH of the rice transgenics and wild type seeds after accelerated ageing treatment. Seeds were imbibed in water for four hours and the resultant water leachate was used for quantification of EC and pH. d) MDA levels, Student t-test at $P < 0.05$ between wild type and transgenic seeds were tested. Different letters above the error bars indicate significance levels, Three biological replications were maintained for experiments with a minimum 10 seeds for biochemical compounds quantification.
